# Response to immune checkpoint inhibition in a meningioma with DNA mismatch repair deficiency

**DOI:** 10.1093/noajnl/vdae092

**Published:** 2024-06-08

**Authors:** Minh P Nguyen, Damian Almiron Bonnin, Kanish Mirchia, William C Chen, Ezequiel Goldschmidt, Steve E Braunstein, Arie Perry, David R Raleigh, Nancy Ann Oberheim Bush

**Affiliations:** Department of Radiation Oncology, University of California San Francisco, San Francisco, California, USA; Department of Neurosurgery, University of California San Francisco, San Francisco, California, USA; Department of Pathology, University of California San Francisco, San Francisco, California, USA; Department of Radiation Oncology, University of California San Francisco, San Francisco, California, USA; Department of Neurosurgery, University of California San Francisco, San Francisco, California, USA; Department of Pathology, University of California San Francisco, San Francisco, California, USA; Department of Radiation Oncology, University of California San Francisco, San Francisco, California, USA; Department of Neurosurgery, University of California San Francisco, San Francisco, California, USA; Department of Pathology, University of California San Francisco, San Francisco, California, USA; Department of Radiation Oncology, University of California San Francisco, San Francisco, California, USA; Department of Neurosurgery, University of California San Francisco, San Francisco, California, USA; Department of Pathology, University of California San Francisco, San Francisco, California, USA; Department of Neurosurgery, University of California San Francisco, San Francisco, California, USA; Department of Radiation Oncology, University of California San Francisco, San Francisco, California, USA; Department of Pathology, University of California San Francisco, San Francisco, California, USA; Department of Radiation Oncology, University of California San Francisco, San Francisco, California, USA; Department of Neurosurgery, University of California San Francisco, San Francisco, California, USA; Department of Pathology, University of California San Francisco, San Francisco, California, USA; Department of Neurosurgery, University of California San Francisco, San Francisco, California, USA; Department of Neurology, University of California San Francisco, San Francisco, California, USA

**Meningiomas are the most common primary intracranial tumors,^1^ and are mostly treated with surgery and ionizing radiation.^2^ A subgroup of meningiomas recur despite multimodal therapy, and medical treatments are actively being investigated for meningiomas that are resistant to standard interventions.^3^ Immune checkpoint inhibitors (ICI) that target interactions between programmed death receptor (PD-1) and programmed death ligand 1 (PD-L1) have revolutionized the treatment of solid tumors.^4^ The efficacy of ICI in meningiomas is under investigation.^5,6^**

Here we present the case of a 45-year-old female who developed headache and diplopia and was found to have an intracranial left sphenoid wing mass on brain magnetic resonance imaging (MRI). She underwent 3 operations over the next 5 years due to serial local progression, with pathology demonstrating a CNS WHO grade 1 meningioma after each resection. After the third surgery, she was treated with intensity-modulated radiotherapy (IMRT) to the left sphenoid wing for a total dose of 54Gy in 30 fractions. A brain MRI 3 years later revealed a third recurrence measuring 1.9 × 3.5 × 3.7 cm (volume 15.50 ml) (Figure 1a, Supplementary Figure 1). She underwent a fourth operation with residual enhancing tumor measuring 1.5 × 2.8 × 3.7 cm (13.14 ml). Pathology demonstrated a CNS WHO grade 2 atypical meningioma with increased mitotic figures, loss of architecture with sheet-like growth pattern and hypercellular, macronucleoli, >4 mitoses per 10 high-power fields (HPF) (>2.5 mitoses per mm^2^), and elevated immunohistochemical (IHC) staining for Ki67 (Figure 1b).

**Figure 1.**
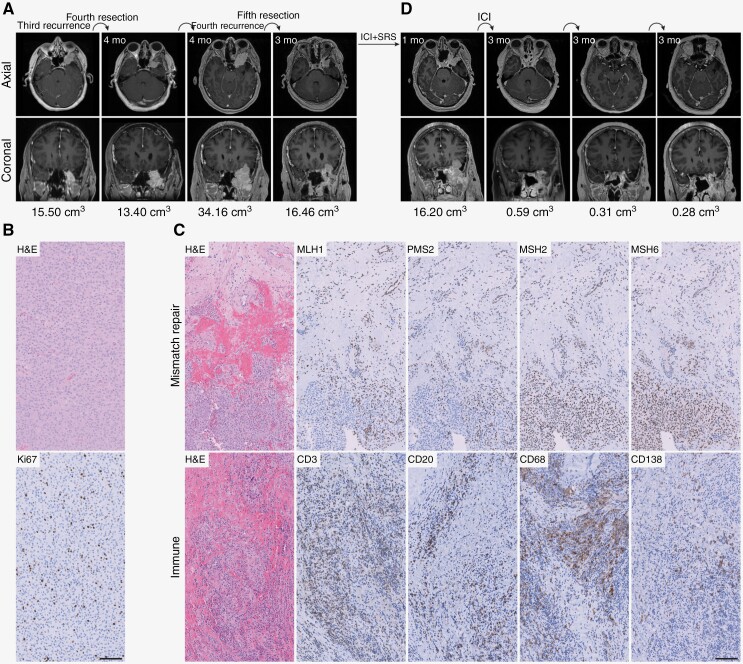
Immune checkpoint inhibition response in a meningioma with DNA mismatch repair deficiency. (a) Serial magnetic resonance imaging prior to immune checkpoint inhibitor treatment. (b) H&E and Ki67 IHC images representative of the fourth and fifth resection specimens. Scale bar: 100 μm. (c) H&E and IHC images for MMR pathway members or for immune cell types representative of the fourth and fifth resection specimens. Scale bar, 100 μm. (d) Serial magnetic resonance imaging after initiation of ICI treatment.

Targeted next-generation DNA sequencing (NGS) of 529 cancer-related genes, select introns, and upstream regulatory regions identified homozygous focal deletion of the *MLH1* gene and over 40 nonsynonymous mutations that were mostly comprised of G>A/C>T transitions and indels causing frameshifts. The estimated somatic mutation burden was 14.4 mutations per megabase and there was instability in 6 out of 85 microsatellite sites tested (7%), consistent with low to moderate DNA mismatch repair (MMR) deficiency.^7^ In support of these findings, there was loss of IHC staining for MLH1 and PMS2, which is degraded when MLH1 is absent, but retained IHC staining for MSH2 and MSH6 (Figure 1c). No mutations were detected in *POLE* or *POLD1* that might confer sensitivity to immunotherapy.^8^ There was no monosomy of chromosome 22q and none of the most commonly encountered pathogenic variants in meningiomas were encountered, including *CDKN2A/B*, the *TERT* promoter, *SMO*, *AKT1*, *PIK3CA*, *PIK3R1*, *TRAF7*, *KLF4*, *SUFU*, *ARID1A*, *SMARCB1*, *BAP1*, *POLR2A*, *SMARCE1*, or *NF2*,^9^ although *NF2* deletion and/or chromosome 22q loss are only seen in 50%–60% of menigniomas.^10–12^ Furthermore, multiple copy number losses were noted, including loss of chromosomes 1p, distal 2p, 3p, 4p, distal 7p, distal 7q, 11p, and 12p. There were also focal inflammatory infiltrates (mostly at the periphery of the tumor) that were comprised of T cells (CD3), B cells (CD20), macrophages (CD68), and plasma cells (CD138) (Figure 1c). The tumor histologic findings and immune profile were thus consistent with a diagnosis of meningioma.

A brain MRI 4 months postoperatively showed a rapid fourth recurrence, measuring 3.5 × 3.0 × 4.6 cm (34.16 ml) with extensive local extension, including abutment of the internal carotid artery, and increased involvement of the superior orbit and orbital apex (Figure 1a). The patient underwent a fifth resection with staged surgical debulking through a left orbitozygomatic craniotomy followed by an endoscopic endonasal approach. Postoperative brain MRI showed residual tumor measuring 16.46 ml. Pathology again revealed an atypical meningioma, CNS WHO grade 2, with 5 mitoses per 10 HPF (3.1 per mm^2^), infiltration of the upper respiratory mucosa and bone, Ki67 labeling index of 15%, and similar MMR and immune IHC staining profiles to prior (Figure 1b, c). Repeat NGS also showed a similar genomic profile, although 25 of 85 microsatellite sites tested (29%) were now unstable.

Postoperatively, the patient enrolled on a clinical trial of ICI and stereotactic radiosurgery for recurrent meningiomas that is currently accruing patients (NCT04659811). She received pembrolizumab (200 mg) 1 month after the fifth resection in conjunction with Gamma Knife stereotactic radiosurgery for a total dose of 25Gy in 5 fractions. A brain MRI 2 weeks after completion of re-irradiation demonstrated stable disease measuring 16.20 ml. Over the next month, she received 2 more pembrolizumab infusions, and MRI 3 months after initiation of ICI demonstrated a significant response, with dramatic reduction in the size of the residual meningioma to 0.7 × 0.7 × 1.0 cm (0.59 ml) (Figure 1d, Supplementary Figure 1). No further treatment was administered, and subsequent brain MRI 3 months later demonstrated further reduction in the size of the residual meningioma to 0.5 × 0.5 × 0.6 cm (0.31 ml) which remained stable after an additional 3 months (0.5 × 0.6 × 0.6 cm; 0.28 ml).

This case of an atypical meningioma with homozygous *MLH1* deletion leading to impaired DNA MMR that was resistant to standard interventions, but which responded to combined ICI and re-irradiation, highlights a genetic scenario conferring unique therapeutic vulnerability. Radiotherapy is associated with slow reduction in meningioma volume by approximately 30% over 2–3 years in those that respond.^2^ The rapid reduction of tumor size and durable response in this case likely suggests vulnerability to ICI in the setting of MMR deficiency. A prior study of meningioma sequencing data found high mutation burden, defined as at least 10 mutations per megabase,^13^ in approximately 2.5% of meningiomas, with a small subgroup of meningiomas showing loss of function mutations in MMR pathway members.^14^ There are currently several ongoing clinical trials studying ICI in meningiomas alone (NCT03173950, NCT03279692, NCT03016091) or in combination with radiotherapy (NCT03604978, NCT02648997, NCT04659811, NCT03267836), all with inclusion criteria that are agnostic to MMR status. One phase 2 clinical trial of pembrolizumab alone in recurrent CNS WHO grade 2/3 meningiomas reported a potential benefit in a subgroup of tumors, but did not report molecular data to define this group.^6^ Impaired MMR and increased mutational burden increases vulnerability to ICI in other tumors through generation of neoantigens.^15^ While cases of meningioma with impaired MMR are rare, 1 previous report described a patient with atypical right frontal convexity meningioma that recurred after multiple surgeries and radiotherapy but responded, dramatically, to nivolumab without radiotherapy.^14^ ICI may, therefore, be an effective therapy for some meningiomas, with or without concomitant radiotherapy. Improved molecular characterization of these tumors is vital to allow for better selection of patients who are most likely to benefit from ICI and other emerging therapeutic strategies for meningiomas that are resistant to standard interventions.

## Supplementary material

Supplementary material is available at *Neuro-Oncology Advances* (https://academic.oup.com/noa).

vdae092_suppl_Supplementary_Figure
