# Supplementary figures and images for: Response to immune checkpoint inhibition in a meningioma with DNA mismatch repair deficiency
Source: Neurooncol Adv. 2024 Jun 8;6(1):vdae092. doi: 10.1093/noajnl/vdae092 (PMC11217898; doi:10.1093/noajnl/vdae092)

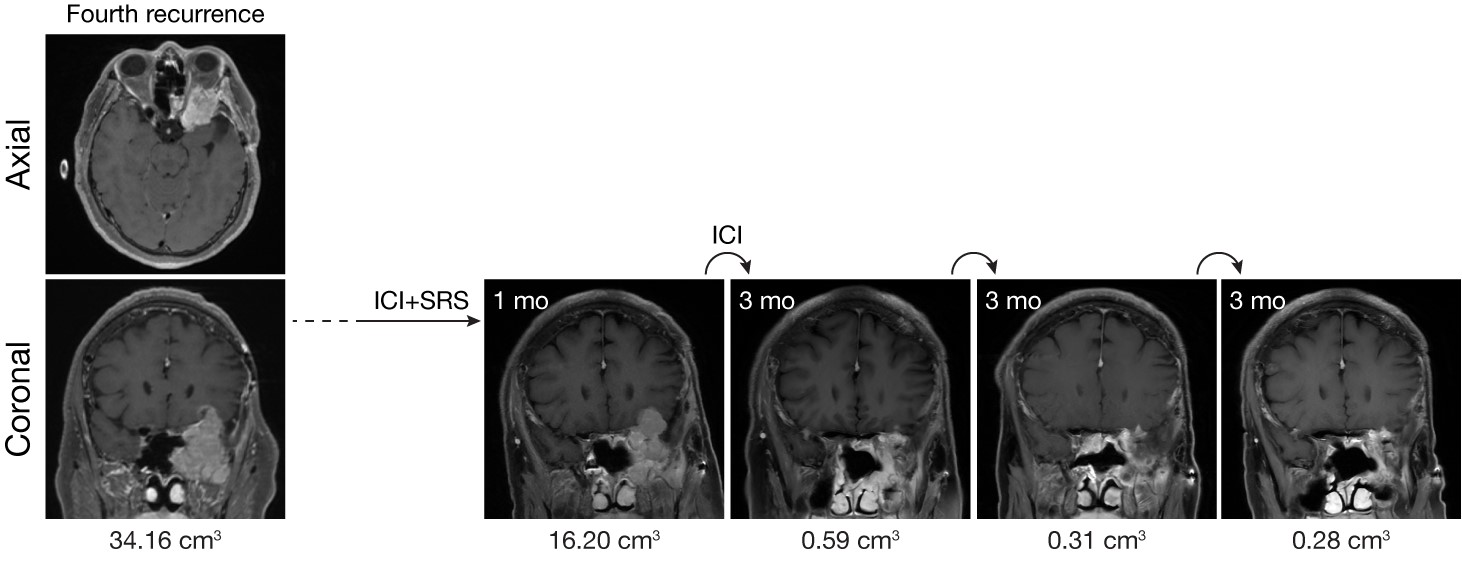

Supplement: vdae092_suppl_Supplementary_Figure [file vdae092_suppl_supplementary_figure.jpeg]
